# Comparison of the response of alternative oxidase and uncoupling proteins to bacterial elicitor induced oxidative burst

**DOI:** 10.1371/journal.pone.0210592

**Published:** 2019-01-10

**Authors:** Ádám Czobor, Péter Hajdinák, Bence Németh, Borbála Piros, Áron Németh, András Szarka

**Affiliations:** 1 Department of Applied Biotechnology and Food Science, Laboratory of Biochemistry and Molecular Biology, Budapest University of Technology and Economics, Budapest, Hungary; 2 Department of Applied Biotechnology and Food Science, Fermentation Pilot Plant Laboratory, Budapest University of Technology and Economics, Budapest, Hungary; Institute for Sustainable Plant Protection, C.N.R., ITALY

## Abstract

Plant UCPs are proved to take part in the fine-tuning of mitochondrial ROS generation. It has emerged that mitochondrion can be an important early source of intracellular ROS during plant-pathogen interaction thus plant UCPs must also play key role in this redox fine-tuning during the early phase of plant–pathogen interaction. On the contrary of this well-established assumption, the expression of plant UCPs and their activity has not been investigated in elicitor induced oxidative burst. Thus, the level of plant UCPs both at RNA and protein level and their activity was investigated and compared to AOX as a reference in *Arabidopsis thaliana* cells due to bacterial harpin treatments. Similar to the expression and activity of AOX, the transcript level of *UCP4*, *UCP5* and the UCP activity increased due to harpin treatment and the consequential oxidative burst. The expression of *UCP4* and *UCP5* elevated 15-18-fold after 1 h of treatment, then the activity of UCP reached its maximal value at 4h of treatment. The quite rapid activation of UCP due to harpin treatment gives another possibility to fine tune the redox balance of plant cell, furthermore explains the earlier observed rapid decrease of mitochondrial membrane potential and consequent decrease of ATP synthesis after harpin treatment.

## Introduction

The first stage of plant-pathogen interaction begins after the contact of pathogen with the plant surface, then it secretes different protein and non-protein effectors called pathogen-associated molecular patterns (PAMPs) [1]. The translocation of PAMPs and the successful suppression of plant immune system that results in the penetration and establishment of the pathogen means that pathogen invasion was successful [2–4]. Parallel a complex signalling network is activated that resulted in the hypersensitive response (HR) and/or the induction of systemic acquired resistance (SAR) [1]. HR involves the activation of programmed cell death (PCD) and the accompanying oxidative burst [5,6]. Oxidative burst is also an early event of the incompatible plant-pathogen interactions [7]. Many enzymatic elements of the plant cells are involved in the generation of excess amount of reactive oxygen species (ROS), hence the activation of plasma membrane-localized NADPH-oxidases [8], cell wall peroxidases [9,10], and apoplastic amine, diamine, and polyamine oxidases [11]. The generation of ROS can be linked to different subcellular organelles, such as the mitochondria [12,13], chloroplasts, and peroxisomes [1,14]. Two different phases of the oxidative burst response can be recognized in incompatible interactions [15,16]. While the first phase is nonspecific and occurs right after the addition of either compatible or incompatible pathogens, the second phase occurs 1.5–3 h after the inoculation and it probably affects only the incompatible plant-pathogen interactions [17]. On one hand ROS generated during the oxidative burst play essential role in the defence again pathogen, since hydroxyl radical, can kill the pathogen directly [18], ROS can behave as secondary messengers through the redox control of transcription factors and establish the interaction with other signalling pathways, such as phosphorylation cascades [19–21], they can also take part in the construction of physical barriers [22,23], furthermore they generate jasmonate-type signalling cyclic oxylipins [24] and phytoalexins, secondary metabolites to arrest pathogen growth [25].

On the other hand the level of ROS must be kept below a certain level otherwise they can cause severe cytotoxicity [26]. This nontoxic level of ROS for their signalling role can be achieved by the fine tuning of ROS production and scavenging pathways [27]. There are two different ways to regulate the amount of ROS in plant cells: 1. by the regulation of their production, 2. by scavenging them via different antioxidant mechanisms [28]. Since mitochondria represent one of the major sources of ROS during stress in plant cells [29] and the mitochondrion is likely an important player in plant PCD, including the HR [30] it has emerged that mitochondrion may be an important early source of intracellular ROS during plant-pathogen interaction, because of the so-called mitochondrial oxidative burst. Indeed, in bacterial elicitor treated *Arabidopsis* cell cultures it was shown that a large and early ROS burst is produced specifically from the mitochondrion, suggesting the electron transport chain (ETC) as the likely source of ROS [31]. This burst of mitochondrial ROS was associated with a decline in mitochondrial membrane potential and cellular ATP levels and the appearance of cytosol-localized cytochrome c. All these events preceded cell death by several hours [31]. At cellular level there are two different ways to regulate the amount of ROS in plant mitochondria: The first way to influence ROS production is to cause or prevent over-reduction of the ETC. The over-reduction of the ETC can be avoided by the means of several unique (plant mitochondria specific) ETC components such as the alternative rotenone resistant NAD(P)H dehydrogenases [32], alternative oxidase (AOX) [33] and the uncoupling proteins (UCPs) which can also be found in animal cells [34].

AOX is the best characterized from the above-mentioned unique plant mitochondrial components. It catalyses the direct oxidation of ubiquinone and reduction of O_2_ to H_2_O [33]. AOX also reduces the energy yield of respiration because it is non-proton pumping and bypasses proton-pumping complexes III and IV. Hence AOX can be called as a “safety electron valve” since one of its key roles is to prevent over-reduction of the ETC and allow the continued operation of glycolysis and the tricarboxylic acid cycle [35]. Accordingly, AOX maintains the redox balance of the ubiquinone pool, thus minimizing the formation of ROS from reduced ubiquinone [36,37]. AOX transcripts and activity are increased in response to pathogen attack [38–41], freezing and chilling [42,43], or low phosphate availability [35,44,45].

Beyond AOX, plant mitochondria contain other “safety valves” in the form of UCPs. UCPs mediate the re-entry of protons–transported by the proton-pumping complexes of the mitochondrial ETC–into the matrix bypassing the ATP-synthetase, hence dissipating the electrochemical proton gradient as heat [34]. At least four different roles were attributed to the UCPs: the generation of metabolic (nonshivering) thermogenesis [46], the control of mitochondrial ROS production [47], response to different stress situations [29], and the regulation of energy metabolism [48]. According to these roles the upregulation of *UCP* genes in plants could be observed in cold [49,50] and heat [51] stress, drought, mechanical (wound) stress and in response to fungi, nematoda and RNA virus induced pathogen attack [52–54]. A mild uncoupling delivered by plant UCPs results in the acceleration of respiration consequently it decreases the generation of superoxide, because on one hand it decreases the tissue O_2_ tension on the other hand it minimizes the steady-state concentration of reduced respiratory components [34]. Not surprisingly superoxide could activate UCP in potato mitochondria [55]. On the base of these observations a possible physiological role for plant UCPs is the fine tuning of mitochondrial membrane potential that is optimal for oxidative phosphorylation with minimal production of ROS to protect mitochondria from oxidative damage [34].

Although the role and activity of AOX was investigated in plant pathogen interaction thoroughly [13,35,39,56–58], the role and activity of the other mentioned "safety valve", plant UCP is unknown in bacterial elicitor induced oxidative burst and HR up to date. Thus, both the expression at RNA and protein level and the activity of plant UCP was investigated in *Arabidopsis thaliana* cell cultures treated by harpin protein from *Pseudomonas syringae* pv. tomato DC3000 (HrpZ_pto_).

## Materials and methods

### Materials

Murashige and Skoog medium, 2,4-dichlorophenoxyacetic acid (2,4-D), kinetin, 2-(N-morpholino)ethanesulfonic acid (MES), triphenil-tetrazolium chloride (TTC), xylenol orange, EDTA, succinate, 4-morpholinepropanesulfonic acid (MOPS), Polyvinylpyrrolidone (PVP-40), hydroxylamine, sulphanilamide, α-naphthylamine, ampicillin, NP40, safranin, Salicylhydroxamic acid (SHAM), kalium-cyanide (KCN), linoleic acid, fatty acid free bovine serum albumin (BSA), luminol, p-Coumaric acid, Carbonyl cyanide-p-trifluoromethoxyphenylhydrazone (FCCP), were obtained from Sigma-Aldrich. ProBond Purification System was purchased from Invitrogen. Amicon Ultra 30K Centrifugal Filter Units were purchased from Merck. IPTG was obtained from Duchefa Biochemie, cytochrome c was purchased from Fluka. Primary and secondary antibodies were purchased from Agrisera Antibodies. All other chemicals were of analytical or HPLC grade, and were purchased from Reanal, Hungary. Pierce BCA Protein Assay Kit, GeneJET Plant RNA Purification Kit, and RevertAid First-Strand cDNA Synthesis Kit were obtained from Thermo Scientific; SensiFAST SYBR No-ROX Kit was purchased from Bioline.

### Plant material

*Arabidopsis thaliana* (ecotype Columbia) suspension cells were grown in culture medium containing 0.44% MS + Gamborg (Sigma-Aldrich); 3% Sucrose; 0.24 μg/ml 2,4-dichlorophenoxyacetic acid; 0.014 μg/ml Kinetin; pH 5.8 in a rotary shaker (120 rpm) at 22°C in the dark. Cells were subcultured weekly by a tenfold dilution [59].

### Harpin production and purification

Harpin (HrpZ_pto_) producing *Escherichia coli* line [60] was a generous gift from Dr. Alen Collmer (Cornell University, Ithaca, NY, USA). The maintenance of the *E*. *coli* cell line and the harpin production were carried out as described earlier by our research group [41]. Harpin protein was purified by a hybrid method using Invitrogen ProBond Purification System, 5 mM MES (2-(N-morpholino)ethanesulfonic acid) (pH 5.8) was used as final elution buffer [61]. The protein quality was verified by SDS-PAGE. The protein concentration was determined by Pierce BCA Protein Assay Kit with BSA standards. Harpin solutions were stored at -20°C.

### Harpin treatments

Harpin treatments were done on 4-day old *Arabidopsis thaliana* cell cultures. HrpZ_pto_ preparation or HrpZ_pto_ preparation and SHAM (Salicylhydroxamic acid) was added to the cells at a final concentration of 150 nM for HrpZ_pto_ and 1 mM for SHAM. The control cells were treated with the same volume of 5 mM MES (pH 5.8) or 5 mM MES (pH 5.8) and SHAM. To test the effect of ethanol, experiments were also carried out using the same amount (1:250 dilution) of ethanol. At the indicated time points, 15 ml of *Arabidopsis thaliana* cells were harvested by vacuum filtration and frozen in liquid nitrogen [62], then stored at -80°C until analysis.

### Cell viability assay

Cell viability was determined by triphenil-tetrazolium chloride (TTC) reduction assay [63]. Briefly, 20 mg of TTC was dissolved in 1 ml of phosphate buffer (50 mM, pH 7.5) and stored in the dark at 4°C until use. *Arabidopsis thaliana* cells (weighted) were transferred to a microfuge tube and washed with 50 mM phosphate buffer (pH 7.5) then re-suspended in 980 μl of the same buffer and supplemented with TTC stock solution (20 μl) at a final concentration of 1.25 mM. The mixture was incubated in the dark for 1 h then it was centrifuged (16,000g, 2 min). The supernatant was discarded, and 1 ml of ethanol was added to dissolve the formed formazan salts. After 12 h of incubation, it was centrifuged (16,000g, 2 min) and the absorbance of the supernatant was measured at 485 nm. Cell viability was normalized to the freshly harvested, vacuum filtrated cell weight.

### Superoxide anion generation assay

The detection of superoxide anion was carried out by the method of Unger et al. [64]. Superoxide was detected by the oxidation of hydroxylamine to nitrite. At the indicated time points, 135 μl of *Arabidopsis thaliana* cells were withdrawn and incubated with 135 μl of Na-phosphate buffer (50 mM, pH 7.8) and 30 μl hydroxylamine (10 mM) in the dark. After 45 min of incubation the samples were centrifuged at 16,000g for 2 min and 100 μl of the supernatant was transferred to a 96-well microtiter plate. To measure the nitrite content of the samples, 100 μl of sulphanilamide (17 mM) and 100 μl of α-naphthylamine (7 mM) was added to each sample and the absorbance was measured at 540 nm. On the base of the following reaction: 2O_2_^·–^+ H^+^ + NH_2_OH → H_2_O_2_ + H_2_O + NO_2_^–^, the concentration of O_2_^·–^was calculated according to the following equation 2[O_2_^·–^] = [NO_2_^–^]. To verify that nitrite production was due to superoxide generated by the cells, a reaction mixture without hydroxylamine was also used.

### Determination of hydrogen-peroxide

The production of hydrogen peroxide was determined by xylenol orange assay [65]. 1 ml of solution A (25 mM FeSO_4_, 25 mM (NH_4_)_2_SO_4_, and 2.5 M H_2_SO_4_) was added to 100 ml of solution B (125 μM xylenol orange and 100 mM sorbitol). 1 ml of *Arabidopsis thaliana* cell suspension was withdrawn and centrifuged at 16,000g for 1 min. 100 μl supernatant was added to 900 μl xylenol orange reagent (1 ml solution A + 100 ml solution B) immediately and incubated at room temperature for 45 min, finally the absorbance was measured at 560 nm. The formation of H_2_O_2_ was verified by the addition of catalase [9].

### Analysis of gene expression

RNA was isolated from *Arabidopsis thaliana* cells by GeneJET Plant RNA Purification Kit. The first-strand cDNA synthesis was performed by Thermo Scientific RevertAid First-Strand cDNA Synthesis Kit. Oligo(dT)18 primer was used. Real-time PCR was performed by Thermo Scientific PikoReal real-time PCR, using SensiFAST SYBR No-ROX Kit with the primer pairs listed in Table 1. The heat program was the following: 95°C/3 min, 30 cycles of 95°C/30 s and 60°C/30 s. Mitosis protein YLS8 was used as housekeeping gene [66].

**Table 1:** The sequence of the applied primers.

Gene	Sequences		References
YLS8	fw:	5'-TTA CTG TTT CGG TTG TTC TCC ATT T-3'	[66]
	rv:	5'-CAC TGA ATC ATG TTC GAA GCA AGT-3'	
AOX1a	fw:	5'-CCG ATT TGT TCT TCC AGA GG-3'	[67]
	rv:	5'-GCG CTC TCT CGT ACC ATT TC-3'	
AOX1b	fw	5’-GGA CAA ACT AGC TTA TTG GAC CGT G-3’	[68]
	rv	5’-TCA TTG CTC TGC ATC CGT ACC-3’	
AOX1c	fv	5’-GGT GGT TCG TGC TGA TGA GG-3’	[68]
	rv	5’-CTT CTT TCA GCT CAT GAC CTT GG-3’	
AOX1d	fv	5’-ACC GTT CAA ACT CTG AAA ATA CCG-3’	[68]
	rv	5’-GCA GCC ACC GTC TCT AGC AA-3’	
AOX2	fv	5’-GGC GAT TTC AAG ATC GGC TC-3’	[69]
	rv	5’-GTT CCA GGC CAA TCC GAT C-3’	
UCP1	fw:	5'-TCT GCT CTT GCT GGT GAT GT-3'	[67]
	rv:	5'-TAC CCA GTG CAC CTG TTG TC-3'	
UCP2	fw:	5'-GGA TTT CAA ACC AAG GAT CG-3'	[67]
	rv:	5'-AGC GCA CTA ACT CCT TCC AG-3'	
UCP3	fw	5’-CAT CTG CTT GCA TTC TCA CTT TGA-3’	[68]
	rv	5’-ACA AAG GCT CTC GTC GGA GG-3’	
UCP4	fw	5’-TGT GCG GTG AAG ACG GTT AAA-3’	[68]
	rv	5’-CAA CAG TGA AAG GAC CTT GCC T-3’	
UCP5	fw	5’-GAC CCA CCC GCT TGA TCT AAT C-3’	[68]
	rv	5’-AAA AGC AAG AGC TGG TCG GAG-3’	

### Isolation of mitochondria

Approximately 100 g of *Arabidopsis thaliana* cells were harvested by vacuum filtration. The cells were homogenized by a grinder. Mitochondria were isolated by differential and Percoll gradient centrifugation as described by Zsigmond et al. [70].

### The determination of AOX and UCP activity

The activity of AOX was determined by a Hansatech Oxygraph at 22°C as described by Zsigmond et al. [71]. AOX activity was determined from 100 μg (protein) of freshly purified intact mitochondria.

The activity of UCP was determined by the method of Pastore et al. [72] and Vercesi et al. [73]. The mitochondrial membrane potential (ΔΨ) was monitored by the fluorescence of safranin (ex.: 495 nm; em.: 586 nm). 100 μg (protein) of freshly prepared, intact mitochondria was added to 2 ml of reaction buffer (150 mM sucrose, 65 mM KCl, 10 mM HEPES, 0.33 mM EGTA, 2.5 μM safranin, pH 7.2) and the fluorescence was followed until the baseline became stable, then succinate was added at the final concentration of 5 mM. 25 μl of fatty acid free BSA (20%) was added, to inhibit the UCP activity. The activity of UCP was calculated from the difference of fluorescence in the presence and in the absence of BSA. To ensure the maximal uncoupled state of mitochondria, FCCP (2 μM)—a potent mitochondrial oxidative phosphorylation uncoupling agent—was added finally. The autofluorescence of every compound was also measured and data were normalized according.

### Protein isolation and western blotting

Protein was isolated in RIPA buffer (150 mM NaCl, 50 mM Tris-HCl, 1% NP40, 0.1% SDS, pH 8) [74] supplemented with Plant protease inhibitor cocktail from Sigma-Aldrich. 20 μg of protein was separated by SDS-PAGE (12% running gel) and blotted to nitrocellulose membrane. To verify the homogeneity of the samples, Ponceau S staining was performed. The membrane was blocked with 5% non-fat dry milk in TBS-Tween buffer (50 mM Tris-HCl, 150 mM NaCl, 0,05% Tween 20, pH 7.9) (1 h), then probed with primary antibodies (listed below) in 1% non-fat dry milk (dissolved in TBS-Tween buffer) overnight at 4°C. The secondary antibody (HRP conjugated, Goat anti-Rabbit by Proteintech Group) was added in 1% non-fat dry milk (dissolved in TBS-Tween buffer) and incubated for 1 h. To visualize the labelled proteins, ECL reagent (100 mM Tris-HCl pH 8.5, 0.2 mM Coumaric acid, 1,25 mM Luminol, 0,1% H_2_O_2_,) was used and the signal was detected by light sensitive film (AGFA). Actin was used as loading control.

The following antibodies were used: anti-UCP (Agrisera Antibodies, AS12 1850), anti-AOX1/2 (Agrisera Antibodies, AS04 054), anti-Actin (Agrisera Antibodies, AS13 2640), HRP-conjugated Goat anti-Rabbit (Proteintech Group, SA00001-2).

The densitometry of Western blot data was carried out by ImageJ software and normalized to actin as the reference protein.

### Other methods

Protein concentration was determined by Pierce BCA Protein Assay Kit with bovine serum albumin as standard, supplied with the kit. All data are expressed as means ± S.D. Statistical analyses (Student’s t test) were performed with SPSS version 13.0.1 (SPSS Inc, Chicago, IL).

## Results

### The effect of HrpZ_pto_ treatment on the viability and ROS generation of Arabidopsis thaliana cells

**Fig 1:**
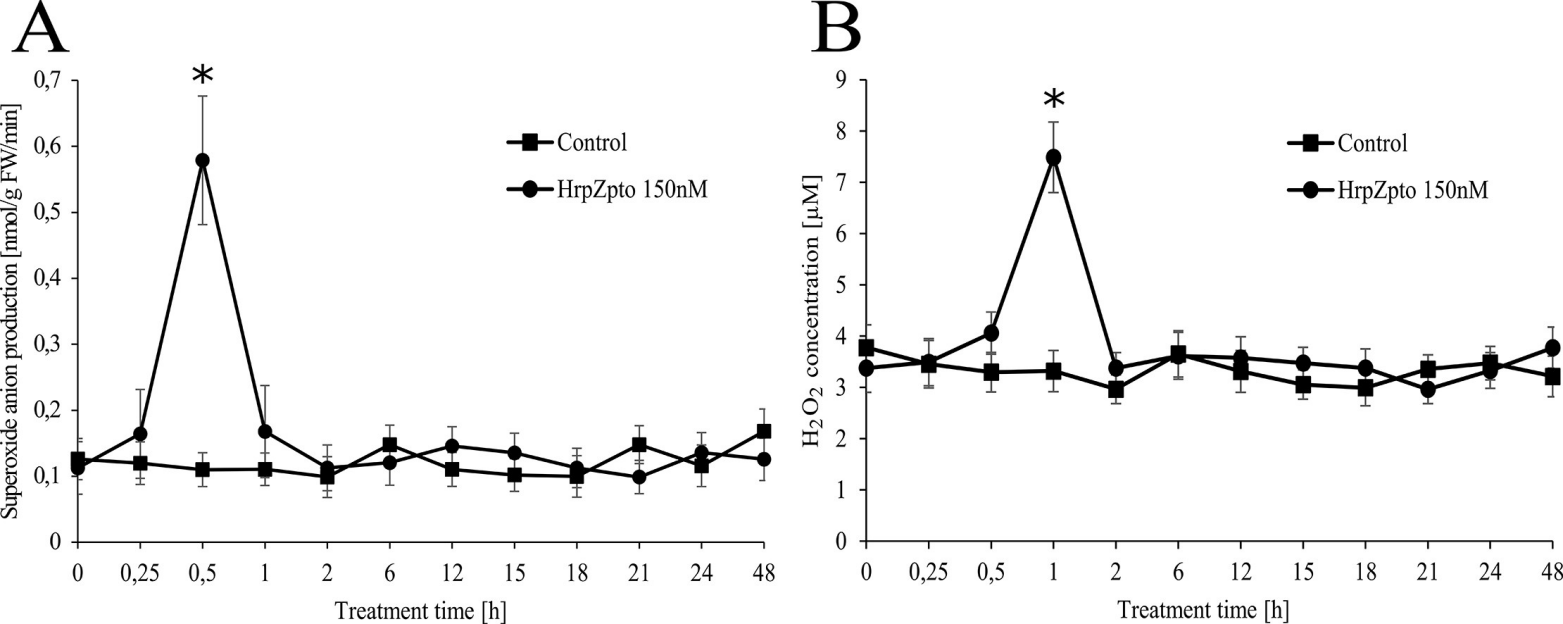
Effect of HrpZ_pto_ treatment on superoxide anion (A) and H_2_O_2_ (B) generation in *Arabidopsis thaliana* suspension cells. *Arabidopsis* suspension cells were treated with HrpZ_pto_ at the final concentration of 150 nM. At the indicated time points, samples were taken, and the generation of superoxide anion was followed by the oxidation of hydroxylamine to nitrite. The H_2_O_2_ content of the samples was determined by xylenol orange assay as described in Materials and methods. Samples, collected from each cell culture before treatments were indicated as zero time point. Value represents mean ± SD from three independent HrpZ_pto_ treatments. (*Asterisk*) Significant difference with respect to control (P<0.05).

The generation of huge amount of ROS is a typical hallmark and an early response to plant–pathogen interaction [1,5]. Accordingly, the typical signs of oxidative burst could be observed in *Arabidopsis thaliana* cells due to HrpZ_pto_ treatment. The level of superoxide anion reached its peak value (5.5-fold of the untreated control) after 30 min (Fig 1A). The maximum level of hydrogen-peroxide could be measured later, after 60 min (Fig 1B). The level of both ROS type decreased quickly, no elevated values could be observed 120 min post-treatment and no further ROS peak could be observed within 48 h of treatment (Fig 1A and 1B panel).

**Fig 2:**
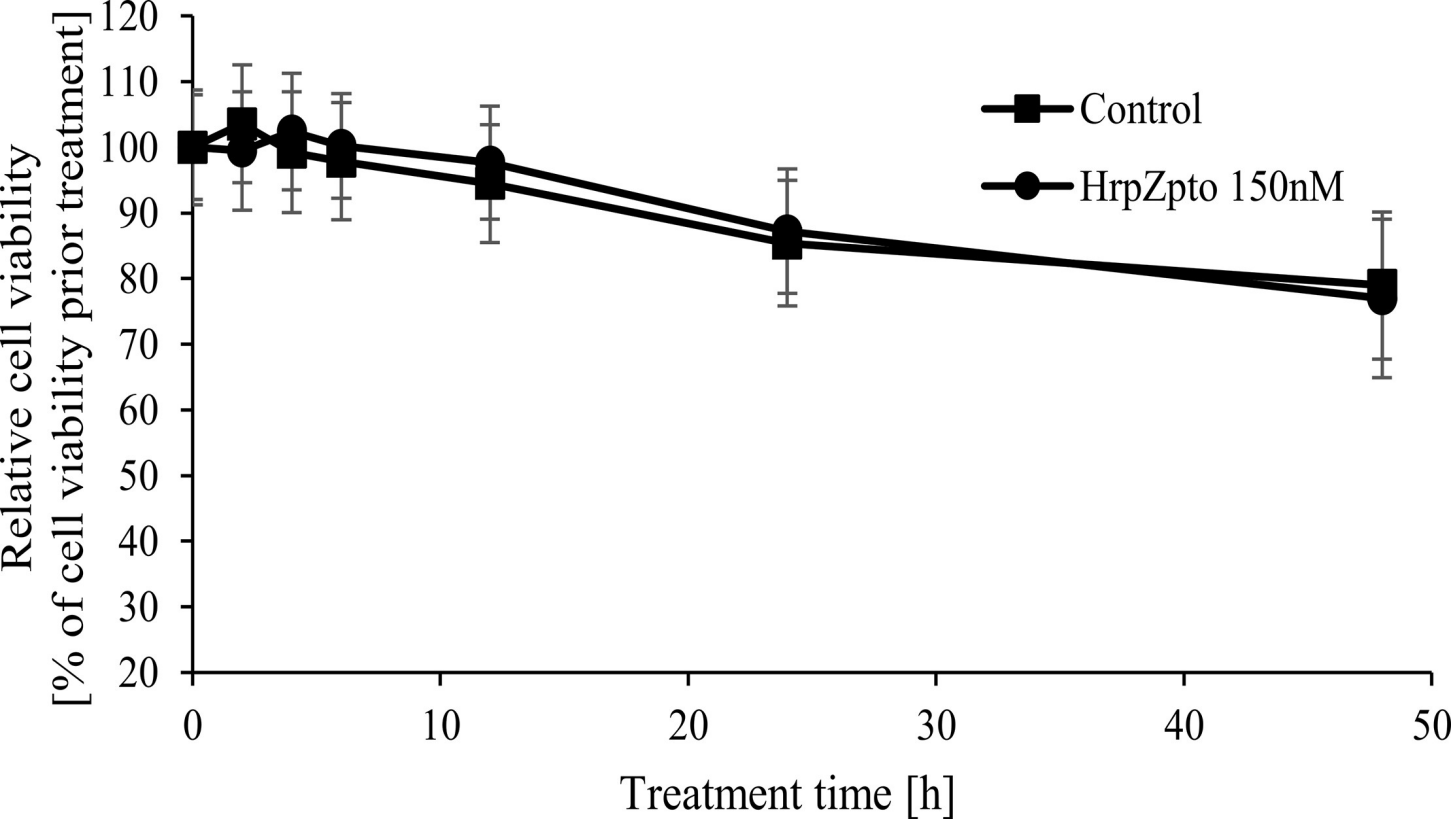
Effect of HrpZ_pto_ treatment on cell viability of *Arabidopsis thaliana* suspension cells. *Arabidopsis thaliana* suspension cells were treated with HrpZ_pto_ at the final concentration of 150 nM. At the indicated time points cell viability was determined by TTC reduction assay as described in Materials and methods. Cell viability of samples collected from each cell culture before treatments was regarded as 100%. Value represents mean ± SD from three independent HrpZ_pto_ treatments.

Similar to our previous results, HrpZ_pto_ treatment caused no enhanced cell death (Fig 2).

### The effect of HrpZ_pto_ on the level and activity of alternative oxidase and uncoupling protein

**Fig 3:**
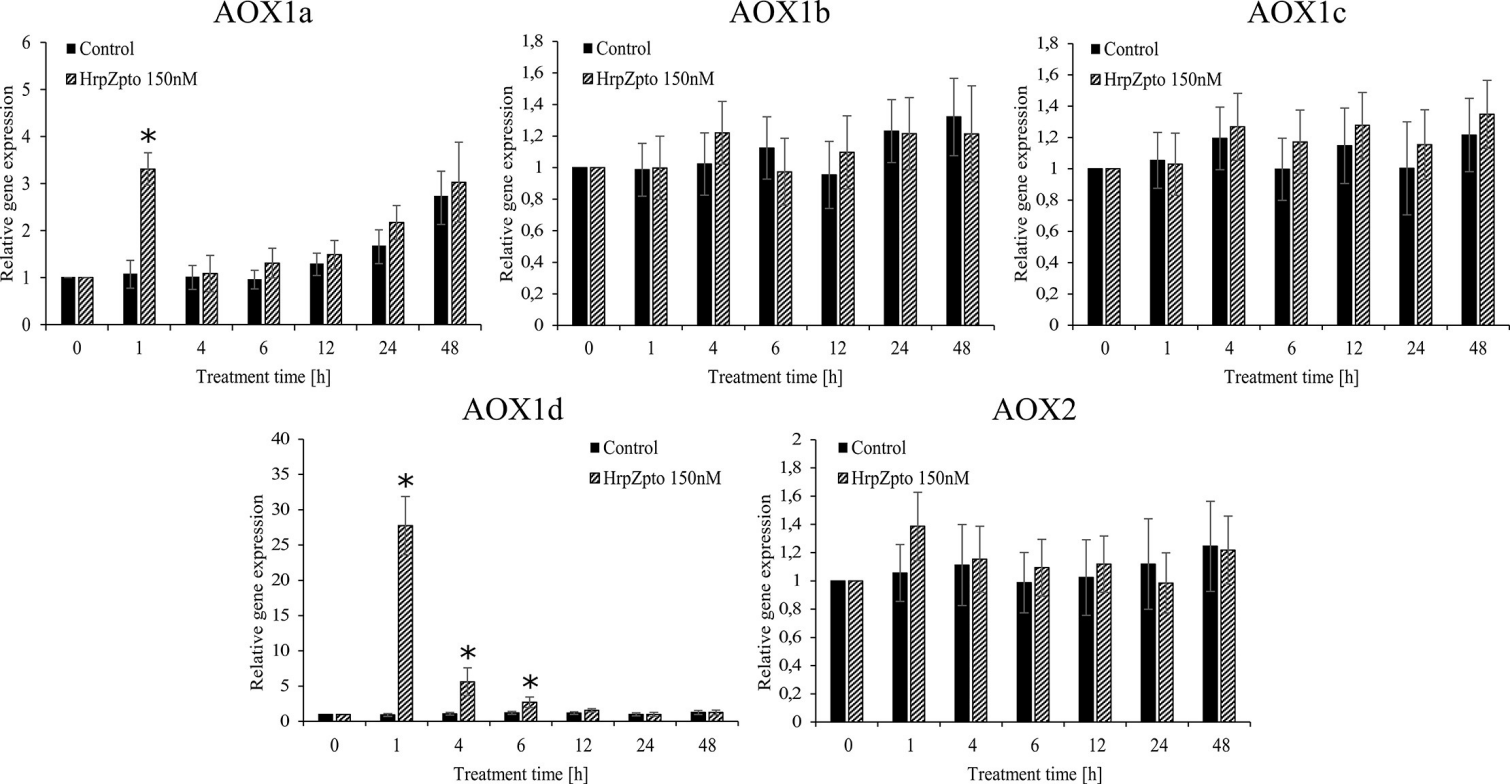
Relative mRNA level of different *AOX* isoforms in control and harpin-treated *Arabidopsis thaliana* suspension cells. *Arabidopsis* suspension cells were treated with HrpZ_pto_ at the final concentration of 150 nM. At the indicated time points, samples were taken, and total RNA was extracted. Quantitative RT-PCR was carried out by using specific primers designed for the coding sequences of *AOX1a*, *AOX1b*, *AOX1c*, *AOX1d*, *AOX2* and mitosis protein *YLS8* (housekeeping gene) genes as described in Materials and methods. The gene expression of the samples collected from each cell culture before treatments, was regarded as 1. Data are expressed as means ± SD from three independent HrpZ_pto_ treatments. (*Asterisk*) Significant difference with respect to control (P<0.05).

The excess ROS generation by mitochondrial electron transfer chain can be avoided by AOX and UCP, thus both the expression and the activity of plant *UCP* and *AOX* was investigated in control and HrpZ_pto_ treated *Arabidopsis thaliana* cells. The RNA level of *AOX1a* elevated together with the level of ROS and reached its maximal value (~3.3-fold of untreated control) after 60 min (Fig 3) then decreased to the basal level after 3 h of harpin treatment (Fig 3). Since *Arabidopsis thaliana* possesses five different *AOX* isoforms (*AOX1a*-*AOX1d* and *AOX2*) [75] the expression of the other four isoforms was also investigated. Similar to the expression of *AOX1a* the expression of *AOX1d* was also significantly elevated due to HrpZ_pto_ treatment. The time course of the elevation also showed similar pattern, it reached the maximal value (~27-fold of untreated control) after 60 min (Fig 3). The expression of the other isoforms was not affected by harpin treatment (Fig 3). Interestingly the level of AOX1a mRNA showed an increasing tendency by aging in both the treated and untreated cells (Fig 3).

**Fig 4:**
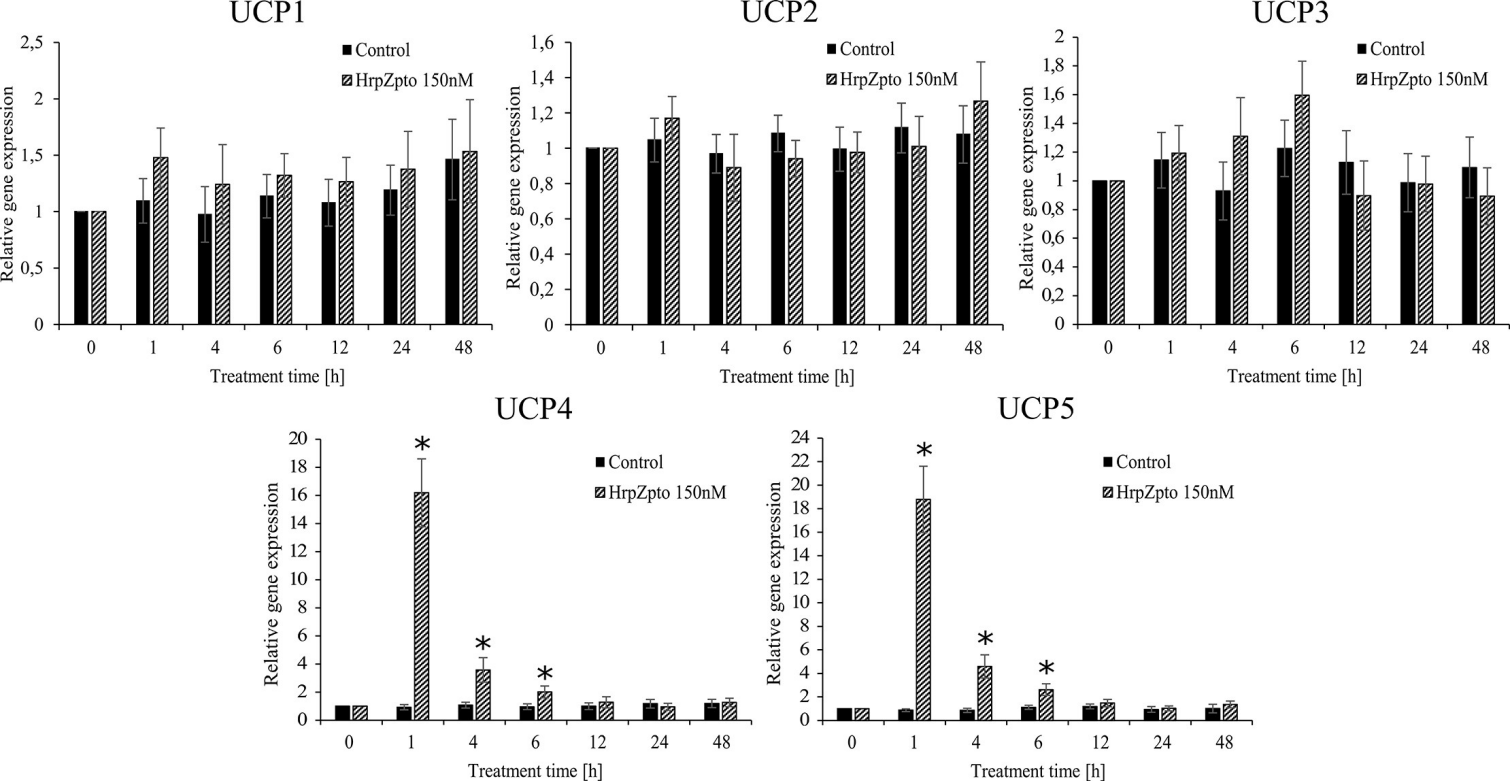
Relative mRNA level of different *UCP* isoforms in control and harpin-treated *Arabidopsis thaliana* cells. *Arabidopsis* suspension cells were treated with HrpZ_pto_ at the final concentration of 150 nM. At the indicated time points, samples were taken, and total RNA was extracted. The quantitative RT-PCR was carried out by using specific primers designed for the coding sequences of *UCP1*, *UCP2*, *UCP3*, *UCP4*, *UCP5* and mitosis protein *YLS8* (housekeeping gene) genes as described in Materials and methods. The gene expression of the samples collected from each cell culture before treatments, was regarded as 1. Data are expressed as means ± SD from three independent HrpZ_pto_ treatments. (*Asterisk*) Significant difference with respect to control (P<0.05).

The expression of *UCP4* and *UCP5* elevated 15-18-fold after 1 h of treatment. Although the expression of both declined quite quickly it was still significantly elevated 4 and 6 h after the treatment (Fig 4). No changes could be observed in the expression of *UCP1*, *UCP2* and *UCP3* due to harpin treatment and the consequential oxidative burst (Fig 4).

**Fig 5:**
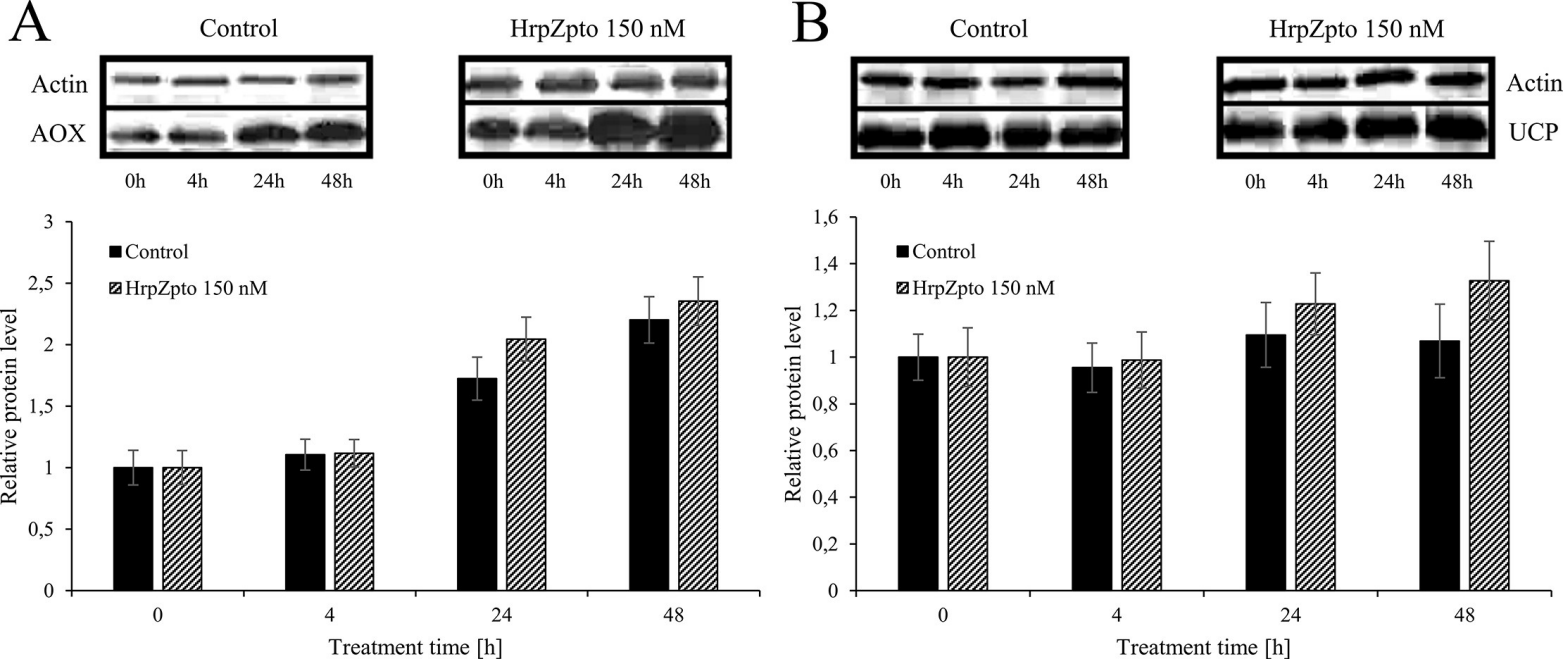
**Relative protein level of AOX (A) and UCP (B) in control and harpin-treated *Arabidopsis thaliana* cells.** *Arabidopsis thaliana* cells were treated with HrpZ_pto_ at the final concentration of 150 nM. At the indicated time points samples were taken, and total protein was extracted. Western blot was carried out by specific antibodies for AOX, UCP and Actin (loading control) as described in Materials and methods. The samples collected from each cell culture before treatments were indicated as time point 0. Data are expressed as means ± SD from three independent HrpZ_pto_ treatments. (*Asterisk*) Significant difference with respect to control (P<0.05).

**Fig 6:**
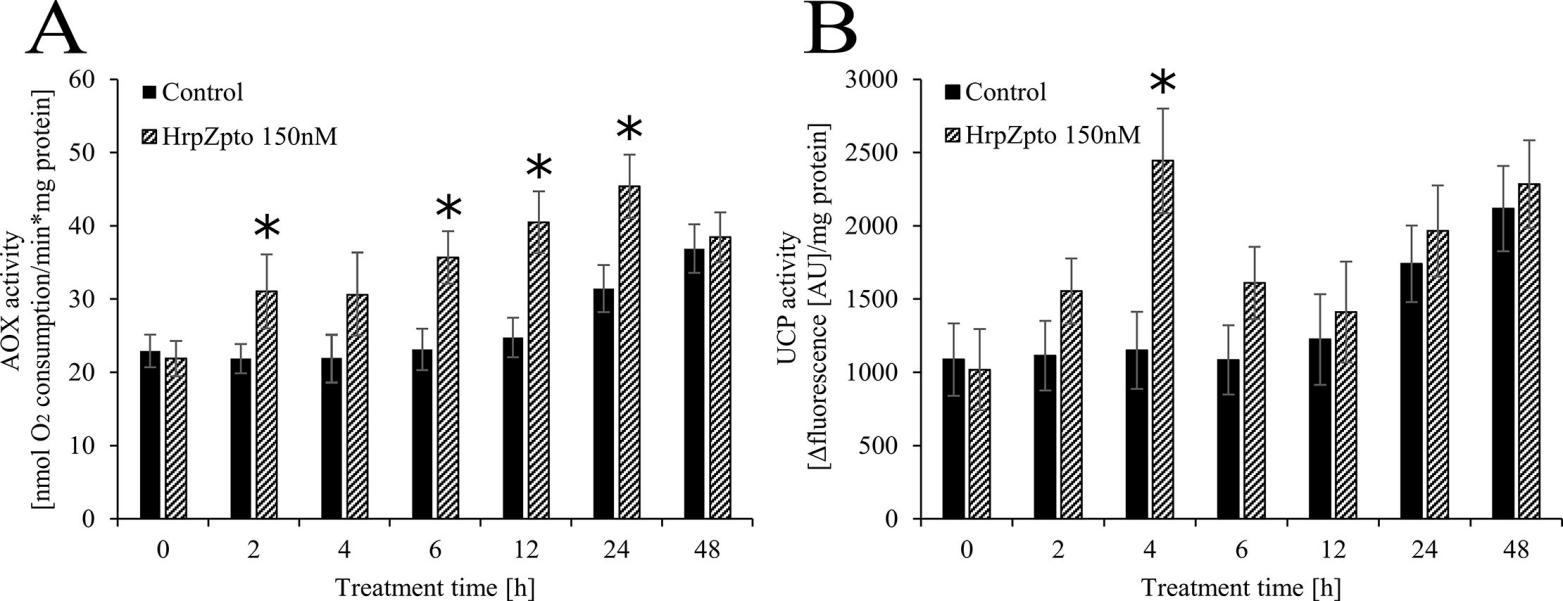
**Effect of HrpZ_pto_ treatments on the activity of AOX (A) and UCP (B) in *Arabidopsis thaliana* cells.** *Arabidopsis thaliana* suspension cells were treated with HrpZ_pto_ at the final concentration of 150 nM. At the indicated time points, samples were taken. The alternative oxidase (AOX) and uncoupling protein (UCP) activities were determined from freshly purified mitochondria from control and HrpZ_pto_-treated *Arabidopsis thaliana* suspension cells as described in Materials and methods. The samples collected from each cell culture before treatments were indicated as time point 0. Data are expressed as means ± SD from three independent HrpZ_pto_ treatments. (*Asterisk*) Significant difference with respect to control (P<0.05).

In the next turn of our experiments the protein levels of AOX and UCP were investigated. Neither the level of AOX, nor the level of UCP changed remarkably due to harpin treatment (Fig 5). According to the gene expression of *AOX* (Fig 3) its protein level also showed increasing tendency by aging, significantly higher protein levels could be observed in the elder cell cultures (5–6 days old) than in the younger ones (3–4 days old) (Fig 5). This phenomenon in the protein and RNA level of UCP could not be observed (Fig 4 and Fig 5). On the contrary to the unchanged protein levels the activity of both AOX and UCP was elevated due to HrpZ_pto_ (150nM) treatment (Fig 6 panel A and B). The activity of AOX responded to the harpin treatment quickly (within 2h). It elevated approximately to 1.4-fold of the activity of the untreated control and reached its maximal value 12h after the harpin treatment with 1.7-fold of the untreated control then fell down to the control value at 48h (Fig 6 panel A).

Similar to the activity of AOX, the activity of UCP responded quite quickly to harpin treatment and to the consequent oxidative burst. A slightly elevated UCP activity (1.4-fold of the untreated control) could be measured as quickly as 2h after the treatment (Fig 6 panel B). UCP activity reached its maximal value 4h after the harpin treatment with 2.1-fold of the untreated control, then it descended to 1.5-fold at 6h (Fig 6 panel B). Finally, no difference could be measured in the UCP activity of harpin treated and non-treated *Arabidopsis* cells after 12h, 24h or 48h of harpin treatment (Fig 6 panel B).

### The effect of HrpZ_pto_ on the rate of respiration

**Fig 7:**
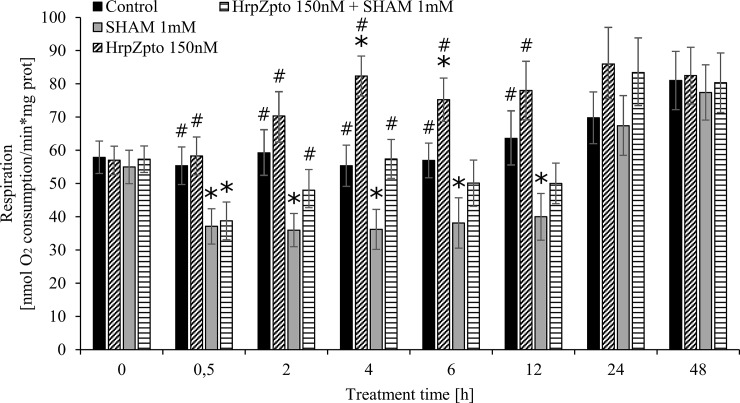
Effect of HrpZ_pto_ treatments on the respiration of *Arabidopsis thaliana* cells. *Arabidopsis thaliana* cells were treated with HrpZ_pto_ (150 nM) or SHAM (1 mM) or both (HrpZ_pto_ (150 nM) and SHAM (1 mM)). At the indicated time points, samples were taken. The respiration of the cells was determined by Clark-type oxygen electrode as described in Materials and methods. The samples collected from each cell culture before treatments were indicated as time point 0. Data are expressed as means ± SD from three independent HrpZ_pto_ treatments. (*Asterisk*) *Significant difference with respect to control; #Significant difference with respect to SHAM treatment, and between control and SHAM treatment indicated by caret mark (^) (P<0.05).

According to the higher AOX and UCP activities, higher respiration rate could be measured in HrpZ_pto_ (150nM) treated cells compared to the untreated controls (Fig 7). In line with the maximal AOX and UCP activity, the respiration reached its maximal rate (~1.5-fold elevation) 4h after the harpin treatment, (Fig 7.). The addition of SHAM (1mM) could decrease the rate of respiration through the inhibition of AOX (Fig 7.). However, the co-treatment of cells by SHAM (1mM) and HrpZ_pto_ (150nM) resulted in higher respiration than SHAM (1mM) treatment alone (Fig 7.) suggesting that a part of the elevation of the respiratory rate resulted from the enhanced activity of UCP in the initial phase (0h-6h) of harpin treatment.

## Discussion

The generation of huge amount of ROS is a typical hallmark and an early response of plant–pathogen interaction [1,5]. This so-called oxidative burst develops almost immediately after the contact of plant cells and pathogen-derived elicitors, such as harpin proteins [31,76]. Although ROS generated during the oxidative burst play essential role in the defense against pathogen, the uncontrolled overproduction of ROS is unequivocally harmful to the plant cell [5]. Approximately 60 min of exposure to H_2_O_2_ was enough to initiate irreversible processes which lead to cell death [77]. The elimination of ROS generated due to pathogen or elicitor protein treatment could clearly mitigate the rate of HR [77,78]. All these observations highlight the importance of the fine-tuning of redox balance in bacterial (elicitor) induced oxidative burst.

Plant UCPs are proved to take part in the fine tuning of mitochondrial ROS generation [34] furthermore it has emerged that the mitochondrion can be an important early source of intracellular ROS during plant-pathogen interaction [31] thus they can play key role in this redox fine-tuning during the early phase of plant–pathogen interaction. On the contrary of this well-established assumption the expression of plant UCPs at both RNA and protein level and their activity has not been investigated in bacterial elicitor induced oxidative burst up to date. To fill this scientific gap, we aimed at the investigation of the level of plant UCPs and their activity in *Arabidopsis thaliana* cells due to bacterial harpin (HrpZ_pto_) treatments.

In concordance with the earlier observations of Desikan et al. [77,79], Reboutier et al. [76] and our group [41] the oxidative burst developed rapidly due to HrpZ_pto_ treatment (Fig 1). The role of plasma membrane-localized NADPH-oxidases [8], cell wall peroxidases [9,10], and apoplastic amine, diamine, and polyamine oxidases [11] was proposed in the generation of excess ROS in oxidative burst. Furthermore, on the base of mitochondrial ROS production in different stresses [29] and the involvement of mitochondria in plant PCD, including the HR [30] mitochondria can also be an important player of oxidative burst. Plant mitochondria also play an essential role in the elimination of ROS [29]. The over-reduction of the elements of plant mitochondrial ETC leads to excess ROS generation. This over-reduction of the ETC can be avoided by the means of several plant mitochondria specific ETC components such as AOX and UCP [29]. The role and the regulation of AOX in plant pathogen interaction is well established [30,35,39,56–58], however almost nothing is known about the regulation and role of plant UCP in bacterial elicitor induced oxidative burst and HR up to date. Thus, both the expression at RNA and protein level and the activity of plant UCP and AOX (as a reference) was investigated in *Arabidopsis thaliana* cell cultures treated by the harpin protein HrpZ_pto_.

According to the earlier observations [80] the RNA level of *AOX1a* elevated together with the level of ROS and reached its maximal value after 60 min of incubation time (Fig 3). Similar increase in *AOX1* transcripts was reported in *Nicotiana sylvestris* after 1 hour of bacterial harpin treatment. The elevation of *AOX1* transcripts was only transient in both cases, since it dropped down to the basal level after 3 h of harpin treatment (Fig 3), [35]. Since *Arabidopsis thaliana* possesses five different *AOX* isoforms (*AOX1a*-*AOX1d* and *AOX2*) [75] the expression of the other four isoforms was also investigated. Similar to the expression of *AOX1a* the expression of *AOX1d* was also significantly elevated due to HrpZ_pto_ treatment. The time course of the elevation of *AOX1d* transcript showed similar pattern (Fig 3). The expression of the other isoforms was not affected by harpin treatment (Fig 3). Our results underline the exceptional role of AOX1a in stress responses. The elevated mRNA level of *AOX1d* suggests that it can be a partner of AOX1a in elicitor induced oxidative burst. This assumption is further strengthened by the observation that *AOX1d* expression was increased in *aox1a* knockout mutants from Arabidopsis (even if it could not compensate fully for the lack of *AOX1a*) [81,82]. Interestingly the level of *AOX1a* mRNA showed an increasing tendency by the elapsed time in both the treated and untreated cells (Fig 3). It is assumed that the aging of cell cultures can be in the background of this phenomenon.

Similar to the expression of *AOX1a* and *AOX1d*, the expression of *UCP4* and *UCP5* elevated 15-18-fold after 1 h of harpin treatment. Although the expression of both declined quite quickly it was still significantly elevated 4 and 6 h after the treatment (Fig 4). There is no data on the expression of *UCP*s in biotic stress, but the expression of *UCP5* was significantly elevated in high-light stress [83,84]. Furthermore, increased transcript level of *UCP4* and *UCP5* could be observed in Cd-exposed *Arabidopsis* seedlings [68]. No changes could be observed in the expression of *UCP1*, *UCP2* and *UCP3* due to harpin treatment and the consequential oxidative burst (Fig 4). In the next turn the protein levels of AOX and UCP were investigated. Neither of the level of AOX, nor the level of UCP changed remarkably due to harpin treatment (Fig 5.). According to the gene expression of AOX (Fig 3) its protein level showed increasing tendency by aging, significantly higher protein levels could be observed in the elder cell cultures (5–6 days old) than in the younger ones (3–4 days old) (Fig 5). This phenomenon in the protein and RNA level of UCP could not be observed (Fig 4 and Fig 5). On the contrary to the unchanged protein levels the activity of both AOX and UCP was elevated due to HrpZ_pto_ treatment (Fig 6 panel A and B). The activity of AOX responded to the harpin treatment quickly. It reached its maximal value 12h after the harpin treatment with 1.7-fold of the untreated control then decreased to the control value at 48h post-treatment (Fig 6 panel A). It is worth to note that very similar elevation and time course pattern of AOX derived respiration could be observed in harpin treated *Nicotiana sylvestris* [35]. On the base of the transcript and protein level it was concluded that AOX activity changes due to harpin treatment were essentially controlled at the posttranslational level [35]. Similar to the activity of AOX, the activity of UCP responded quite quickly to the harpin treatment and to the consequent oxidative burst. It reached its peak value 4h after the harpin treatment (Fig 6 panel B). The transcript level of UCP4 and UCP5 increased several fold due to harpin treatment and the consequential oxidative burst. We found that the non-stressed basic transcript level of UCP5 is approximately the 6% of the transcript level of "main" uncoupling protein, UCP1. As a consequence of the harpin treatment, it elevated approximately 18-fold, hence it can commensurate with the level of UCP1 (Fig 4). Unfortunately, the antibody used for the determination of UCP protein level is specific only for UCP1 and UCP2 [85] and does not bond to UCP4 or UCP5. Hence the protein level of UCP4 and 5 could not be determined. The elevated activity of UCP due to harpin treatment (Fig 6) suggests that the activity of UCP is regulated at transcriptional level or at transcriptional and post-translational levels in biotic stress. The latter assumption is supported by the observation that plant UCP activity is enhanced by ROS or hydroxynonenal [86]. This way the increased superoxide-anion level generated by the HrpZ_pto_ provoked oxidative burst could elevate the activity of UCP both directly and indirectly via the generation of 4-hydroxy-2- trans-nonenal [34].

According to the higher AOX and UCP activity higher respiration rate could be measured in HrpZ_pto_ treated cells compared to the untreated controls (Fig 7). Parallel with the maximal AOX and UCP activity, the respiration reached its maximal rate (~1.5-fold elevation) 4h after the harpin treatment, (Fig 7.). Elevated UCP and AOX activity was described to accompany by higher oxygen consumption and limited superoxide-anion generation [34,87,88]. The addition of SHAM could decrease the rate of respiration by the inhibition of AOX (Fig 7.). However, the co-treatment of cells by SHAM and HrpZ_pto_ resulted in higher respiration than SHAM treatment alone (Fig 7.) suggesting that a part of the elevation of the respiratory rate resulted from the enhanced activity of UCP.

The present experiments demonstrate for the first time that the transcript level of *UCP4* and *UCP5* and the activity of UCP are elevated due to biotic stress. Our results also reinforced the earlier observations on the involvement of plant mitochondria in harpin induced oxidative burst [31]. The role of mitochondria in harpin induced oxidative burst is further strengthened by the activation of AOX due to harpin treatment [31] (Figs 3 and 5 and 6). AOX as a part of the mitochondrial ETC can prevent the overreduction of the ubiquinol pool and reduce the mitochondrial generation of ROS. The elevation of the transcript level and activity of AOX in plant pathogen interaction was documented several times [13,35,39,56–58]. Our results presented here help to understand the earlier observation of Krause and Durner [31]. In their pioneer work they found that harpin-induced defence responses are associated with accumulation of mitochondrial ROS and NO, and specifically with altered mitochondrial functions such as mitochondrial ROS production, and the decrease of mitochondrial membrane potential. The elevated level of *UCP4*, *UCP5* transcripts and UCP activity explain the earlier observed rapid decrease of mitochondrial membrane potential and consequent decrease of ATP synthesis after harpin treatment [31]. The activation of this "safety valve" can contribute to the avoidance of the generation of H_2_O_2_ at a harmful level [87,89]. This way, the activation of UCP can prevent the plant cells from further superoxide-anion generation thus from fatal oxidative damage [31,90] in harpin induced oxidative burst. The quite rapid activation of UCP due to harpin treatment, described by our group herein, gives another possibility to fine tune the redox balance of plant cell.

## Supporting information

S1 File Data_set_elicitor. Data set of all experiments.
(XLSX)
